# A Potential Link between the C5a Receptor 1 and the β_1_-Adrenoreceptor in the Mouse Heart

**DOI:** 10.1371/journal.pone.0146022

**Published:** 2016-01-04

**Authors:** Kuan Hua Khor, Tyson A. Moore, Ian A. Shiels, Ristan M. Greer, Thiruma V. Arumugam, Paul C. Mills

**Affiliations:** 1 School of Veterinary Science, The University of Queensland, Gatton, Queensland, Australia; 2 School of Biomedical Sciences, The University of Queensland, St Lucia, Queensland, Australia; 3 Faculty of Veterinary Medicine, Universiti Putra Malaysia, UPM Serdang, Selangor, Malaysia; Massachusetts Institute of Technology, UNITED STATES

## Abstract

**Purpose:**

Inflammation may contribute to the pathogenesis of specific cardiovascular diseases, but it is uncertain if mediators released during the inflammatory process will affect the continued efficacy of drugs used to treat clinical signs of the cardiac disease. We investigated the role of the complement 5a receptor 1 (C5aR1/CD88) in the cardiac response to inflammation or atenolol, and the effect of C5aR1 deletion in control of baseline heart rate in an anesthetized mouse model.

**Methods:**

An initial study showed that PMX53, an antagonist of C5aR1 in normal C57BL6/J (wild type, WT) mice reduced heart rate (HR) and appeared to have a protective effect on the heart following induced sepsis. C5aR1 knockout (CD88-/-) mice had a lower HR than wild type mice, even during sham surgery. A model to assess heart rate variability (HRV) in anesthetized mice was developed to assess the effects of inhibiting the β_1_-adrenoreceptor (β_1_-AR) in a randomized crossover study design.

**Results:**

HR and LF Norm were constitutively lower and SDNN and HF Norm constitutively higher in the CD88-/- compared with WT mice (*P*< 0.001 for all outcomes). Administration of atenolol (2.5 mg/kg) reduced the HR and increased HRV (*P*< 0.05, respectively) in the wild type but not in the CD88-/- mice. There was no shift of the sympathovagal balance post-atenolol in either strains of mice (*P>* 0.05), except for the reduced LF/HF (Lower frequency/High frequency) ratio (*P*< 0.05) at 60 min post-atenolol, suggesting increased parasympathetic tone of the heart due to the effect of atenolol administration. The HR of the WT mice were lower post atenolol compared to the CD88-/- mice (*P =* 0.001) but the HRV of CD88-/- mice were significantly increased (*P*< 0.05), compared with WT mice.

**Conclusion:**

Knockout of the C5aR1 attenuated the effect of β_1_-AR in the heart, suggesting an association between the β_1_-AR and C5aR1, although further investigation is required to determine if this is a direct or causal association.

## Introduction

An inflammatory response is a common feature of many cardiovascular diseases including hypertrophic cardiomyopathy [1,2,3], congestive heart failure [4] and myocarditis. Inflammation is associated with increased circulating plasma catecholamine activity [5], which can be modified by treatment with β_1_-adrenoreceptor (β_1_-AR) antagonists. These drugs are beneficial in experimental models of sepsis [6], myofibrillar remodeling in congestive heart failure [7] and myocarditis [8].

Sympathetic over-activity during cardiovascular disease may be characterized by an increase in HR and correspondingly lower heart rate variability (HRV) [2,9,10]. HRV reflects the dynamic interplay between the multiple physiologic mechanisms, and is a measure of the instantaneous HR and R-R intervals (intervals between QRS complexes of normal sinus depolarization) [11,12]. HRV may be an important prognostic indicator in cardiovascular disease [13]. Primary therapy of cardiovascular disease includes β-blockers, such a propanolol, metoprolol and atenolol [14], which target either the β_1_-AR (cardioselective antagonist) or β_1_-and β_2_-AR (non-selective antagonist), resulting in beneficial negative inotropic and chronotropic effects.

Beta blockers do not treat the primary cause of the cardiovascular disease. The effect decreases over time, necessitating altered dose rates and/or medication [14]. It is uncertain whether a decreasing effect of β_1_-AR antagonists represents a receptor effect, such as tachyphylaxis, and/or is due to disease progression.

A link between inflammatory disease and adrenergic activity has been suggested following a study investigating α-adrenoreceptors (α-AR) and a potent inflammatory mediator, the complement 5a (C5a) fragment [15] and this link may extend to β_1_-ARs. Furthermore, while basal levels of C5a may have a role in normal cardiac physiology [16], excessive C5a can damage the heart [17,18], while β_1_-AR antagonists are cardioprotective by inhibiting the expression of chemokines during severe sepsis [6].

The role of the complement 5a receptor 1 (C5aR1) in the cardiac response to inflammation and atenolol treatment is unknown. Using wild type (WT) and C5a knockout (CD88-/-) mouse models, we aimed to:

- Assess the role of C5a in the heart rate response to stress with sepsis. We hypothesized that WT mice with pharmacologic blockade of the C5aR1 would exhibit a smaller heart rate in response to sepsis induced by gut ischemia-reperfusion than placebo treated mice;
- Evaluate any protective effect of C5aR1 blockade on survival in conditions of sepsis;
- Assess the role of C5a in the heart rate response to stress (sham surgery) in the absence of sepsis. We hypothesized that CD88-/- mice would exhibit a smaller heart rate response to sham surgery than WT mice;
- Assess the effects of atenolol (a cardioselective β_1_-AR antagonist) on heart rate, HRV and time- and frequency- domain measures of HRV. We hypothesized that heart rate and HRV response to anesthesia and atenolol treatment would differ in CD88-/- mice compared with WT placebo treated mice.

## Material and Methods

We conducted three controlled studies in mice to address these aims and hypotheses. The studies were approved by the animal ethics committee of The University of Queensland (AEC approval number SBMS/085/09).

### Animals

Mice were all male, aged 8–10 weeks old with a mean weight of 25.7 ± 2.9 (range, 20.4–31.8) g. Wild type (WT) mice were C57BL6/J, the strain most widely used for HRV [19,20,21] and myocardial infarction [22] studies. C5a knockout mice were deficient in complement C5aR1, specifically CD88, on a C57BL6/J background (CD88-/-) [16,18].

All mice were allowed *ad libitum* access to standard rodent chow (Special Feed, Glen Forest, WA, Australia), sunflower seeds and water, and housed individually in plastic cages maintained in a facility with a temperature of 24 ± 2°C and a 12 hr light/dark cycle.

### Anesthesia

Animal were anesthetized with 4.0% isoflurane (Delvet Pty. Ltd., Seven Hills, NSW, Australia) in 100% oxygen in an induction chamber then transferred and maintained with 1.25% isoflurane in 100% oxygen delivered by a face mask. Depth of anesthesia was assessed regularly by response to the pedal reflex. All mice were administered 0.9% sodium chloride (5 mL/kg/hr, IP) and secured in dorsal recumbency on a heating pad using adhesive tape.

### Study 1: Effects of Septicemia (Gut Ischemia-Reperfusion) and Inhibition of C5aR1

This pilot study was used to determine if the C5a molecule was involved in heart rate changes during sepsis induced by gut ischemia-reperfusion, by comparing the heart rate response in mice with pharmacological blockade of the C5aR1 to that of placebo-treated mice.

Each mouse was fasted for a period of 15 hr with *ad libitum* access to water prior to experiment.

The WT mice were allocated to pre-treatment with either placebo (n = 9) or a C5aR1 inhibitor, PMX53 [23,24] (n = 7). PMX53 was given intra-peritoneal at 1 mg/kg (in 5% glucose water) [25] 1 hr before the induction of ischemia. Placebo group mice were pre-treated with a similar volume of 0.9% intra-peritoneal sodium chloride.

The abdomen of each mouse was shaved and surgically prepped before a midline incision was made. The anterior mesenteric artery was located and a nylon suture threaded through a 40 mm section of fine rubber tubing was passed under the artery and back through the tubing. Ischemia was induced by tugging the suture back into the tubing and occluding the artery for 30 min. The suture was then removed (reperfusion) and the abdomen was closed using surgical glue. The HR in each mouse was recorded and measured (over a 5 min period every 15 min) from time 0 to 5 hr in addition to clinical observation for 5 hr before euthanasia via cervical dislocation while under anesthesia.

Mice exhibiting metabolic instability, as reflected by increased or decreased respiratory rate or clinically important changes in heart rate, were euthanased immediately such signs were observed, prior to the end of the experiment at 5 hr.

### Study 2: HR in Sham-Operated Anesthetized Mice with and without the C5aR

In study 2, we compared changes in HR in anesthetized WT and CD88-/- mice undergoing sham surgery without induction of sepsis. Two groups of mice, WT (n = 9) and CD88-/- (n = 4) were anesthetized and prepared as per Study 1, although the anterior mesenteric artery was not occluded at any stage. The mice were monitored for 2.5 hr with HR (measured over a 5 min every 15 min) recorded from time 0 to 2.5 hr. At the termination of the experiment, all the mice were euthanased via cervical dislocation whilst under anesthesia.

### Study 3: Cardiac Response to Atenolol in WT and CD88-/- Mice

Following study 2, to better characterize the cardiovascular changes observed, a model to monitor HRV was developed since this had not been standardized in anesthetized mice [26]. We compared the effects of atenolol compared with placebo, in anesthetized WT and CD88-/- mice.

The WT (n = 10) and CD88-/- (n = 10) mice were allocated to receive either atenolol or placebo in a randomized crossover study design with a 7-day washout period. Mice were first anesthetized (described above) and allowed to stabilize for 15 min before baseline HR measurements were recorded for 30 min, then treated with either atenolol (Tenormin^®^, 2.5 mg/kg IP; Astra Zeneca UK Ltd., Macclesfield, Cheshire, United Kingdom) [27] or placebo (0.9% sodium chloride, IP in equal volume to atenolol).

**Fig 1:**
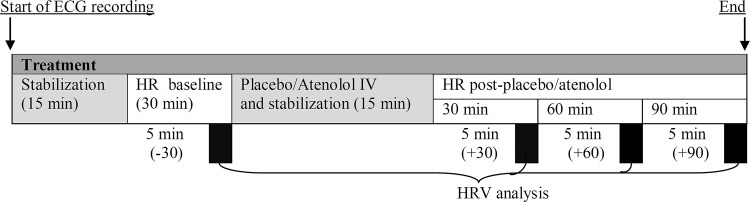
Timeline of the experimental study and the respective time point of data collected for analysis in Study 3.

HR measurements were recorded for a further 90 min and the mice were then permitted to recover from anesthesia. One week later, the mice were again anesthetized and the alternative treatment administered (Fig 1). At the end of the experiment, the mice were euthanased via cervical dislocation whilst under anesthesia.

All the machines and software used to obtain and record HR were sourced from ADInstrument Pty. Ltd. (Bella Vista, NSW, Australia). Lead II ECG was recorded with subcutaneous wire electrodes applied to paw pads on the right forelimb and right hind limb of each mouse. An earth electrode was attached to the other hind limb. Modified electrode clamps were attached to the subcutaneous wire electrodes, secured with tape and connected to an animal bioamplifier (ML136 Animal Bio Amp), then recorded on a PowerLab Data Recorder. Using the acquisition software (LabChart^®^7.0 Pro), the experimental data were continuously recorded in real time with an analog-to-digital conversion data acquisition system connected to a personnel laptop.

### HRV Data Acquisition and Processing

The observation of HR and HRV (time- and frequency-domain measures) were investigated, respectively. An ECG segment recorded free from artifacts was selected at each specific experimental time point for analysis as follows: The HR baseline (-30) and HR post any pharmacological intervention (+30, +60 and +90 min) was acquired over a 5 min period at the end of the 30 min (baseline) and end 30, 60 and 90 min after any pharmacological intervention (see Fig 1).

The segments of raw ECG trace identified were manually inspected to ensure a good quality ECG signal. Each segment was then subjected to HRV analysis using a HRV extension module of LabChart^®^7.0 Pro software. The analytical methodologies used were modified based on previous report adopted in conscious mice [20,26] and cats [2,28]. The modified analytical methodologies established in anesthetized cats [2] was used in this study for acquisition of HRV data. The mean/median of heart rate (HR_mean_/HR_median_) was calculated as the mean/median of the sequence of the reciprocals of the inter-event times.

### HRV Analysis

HRV was quantified and analyzed with the use of standard time- and frequency-domain techniques [11]. Data from the acquisition software were exported and transferred to a Microsoft Excel 2007 spreadsheet for calculations. Five selected time- and frequency-domain measures of HRV were averaged.

#### Time-domain measures

The standard deviation of all normal R-R intervals (SDNN) was calculated directly from the sequence of inter-event times.

#### Frequency-domain measures

Using the R-R interval times series, an R-R interval tachogram was constructed and analyzed as described previously [2]. The cut off frequencies, previously determined to be accurate for mice [20,26], were used to divide the signal into three major components: very low frequency (VLF < 0.15 Hz), low frequency (LF 0.15–1.5) and high frequency (HF 1.5–5 Hz) bands. The squared magnitudes of the discrete Fourier transform of the segments were averaged to form the power spectral density (TP_power_) in ms^2^. The LF Norm, HF Norm, and LF/HF ratios were also calculated.

### Statistical Analysis

All statistical analyses were performed using SPSS 16^®^ (SPSS Inc., Chicago, IL, USA). The level of statistical significance was set at a *P-*value< 0.05. All data distributions were checked for normality.

#### Study 1 and 2

**Fig 2:**
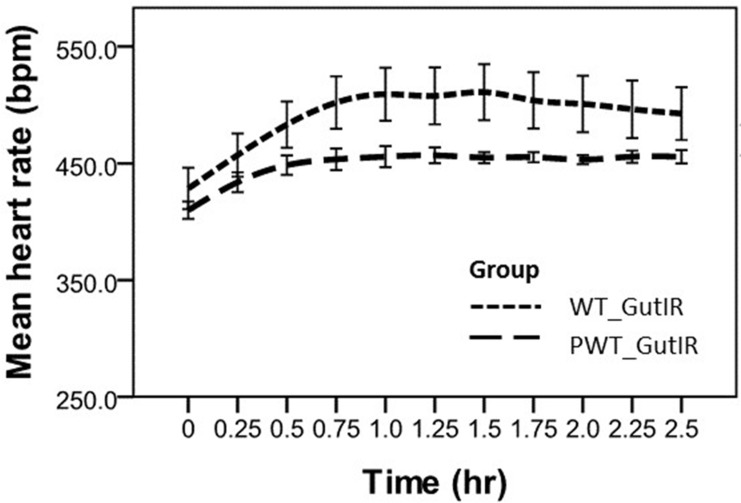
Mean heart rate measurements versus time collected post-gut ischemia reperfusion surgery under anesthesia. Data are presented as the mean ± SEM (error bar). Placebo group; PMX53 group; bpm, beats per minutes; hr, hours. Mean heart rate in the PMX53 group mice was significantly lower (*P*< 0.05) compared to the placebo group mice at each corresponding time point.

**Fig 3:**
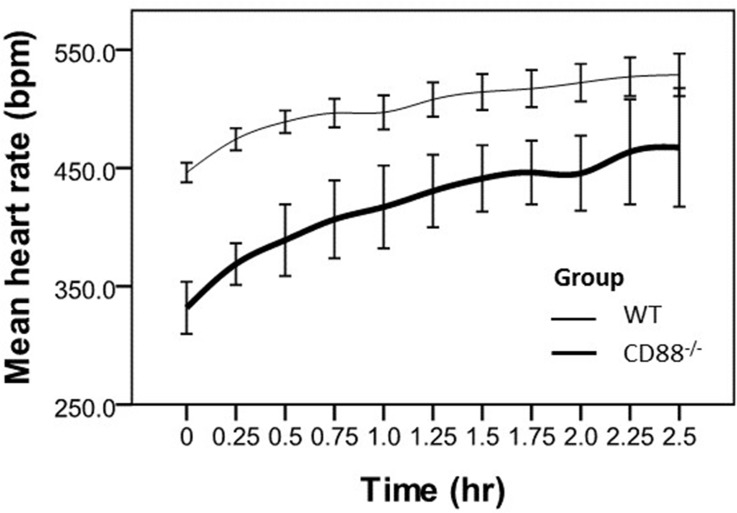
Mean heart rate over time in WT and CD88-/- mice. Data are presented as the mean ± SEM (error bar). WT & CD88-/- mice; bpm, beats per minutes; hr, hours. * Mean HR inCD88-/- mice was significantly lower (*P*< 0.05) compared to the WT mice at each corresponding time point.

The HR at each time point was expressed as mean ± standard deviation (SD) unless otherwise indicated. The differences of the HR_mean_ from mice of both studies (between the placebo and PMX53; and between WT and CD88-/- mice, Figs 2 & 3, respectively) prior to abdominal surgical intervention were compared using two-sample *t-*test. The repeated measurements of mean HR in both studies were recorded in anesthetized mice for up to 5 hr and 2.5 hr, respectively. Comparison was then made between the two strains of mice using a generalized linear model (SPSS genlin) which was adjusted for baseline (beginning of surgical intervention, time = 0 hr).

#### Study 3

Heart rate, SDNN, LF Norm, HF Norm and LF/HF ratio were assessed for normality and summary statistics expressed as mean ± SD or median, interquartile range (IQR), as appropriate, unless otherwise stated. General linear models were used to adjust for baseline heart rate and account for the repeated measures design and consequent correlation between time measurements for individual mice [29]. The primary outcome measures were the difference in response to atenolol, compared with placebo, in WT and CD88-/- mice for HR, SDNN, LF Norm and HF Norm over time. These were compared at four specific time points (1, -30 min; 2, 30 min; 3, 60 min; 4, 90 min) to facilitate comparisons between the relevant variables. Any interaction between mouse strain and time was assessed. The differences in absolute measures between the two strains were also assessed. For each outcome, a number of models were explored, to determine the best fitting model for the effect of drug treatment and mouse strain.

## Results

### Study 1

Mean baseline heart rate prior to surgical intervention differed between the two groups of mice (placebo group 476 ± 45 bpm vs PMX53 group, 439 ± 29 bpm, *P*< 0.0001). Following induction of sepsis, the mean heart rate was lower in the PMX53 group than in the placebo group (391 ± 8 bpm vs. 404 ± 16 bpm, *P*< 0.0001) at time 0 hr. The heart rate increased from baseline to plateau at 0.75 hr for both groups (*P*< 0.0001) with a heart rate increasing more rapidly in the placebo group, (*P* = 0.02, for interaction) (Fig 2).

In the PMX53 group, mean heart rate remained constant and the mice metabolically stable until the end of the experiment at 5 hr. Mice in the placebo group experienced a gradual reduction in heart rate. Five of the 9 mice became metabolically unstable and were euthanased prior to the 5 hr end of the experiment (*P* = 0.10 for difference in survival, Fisher's exact test). The approximate mean survival time for the placebo group mice was 3.75 hr.

### Study 2

Baseline mean heart rate prior to surgical intervention differed between the two groups of mice (WT, 501 ± 29 bpm vs CD88-/-, 409 ± 22 bpm, *P*< 0.0001). Following surgical intervention, the mean heart rate was lower in the CD88-/- compare to the WT mice (325 ± 23 bpm vs. 429 ± 6 bpm, *P*< 0.0001) at time 0 hr. Heart rate increased over the 2.5 hr in both groups (*P*< 0.0001) with no difference in the rate of increase between the groups, *P* = 0.16, for interaction (Fig 3).

### Study 3

**Fig 4:**
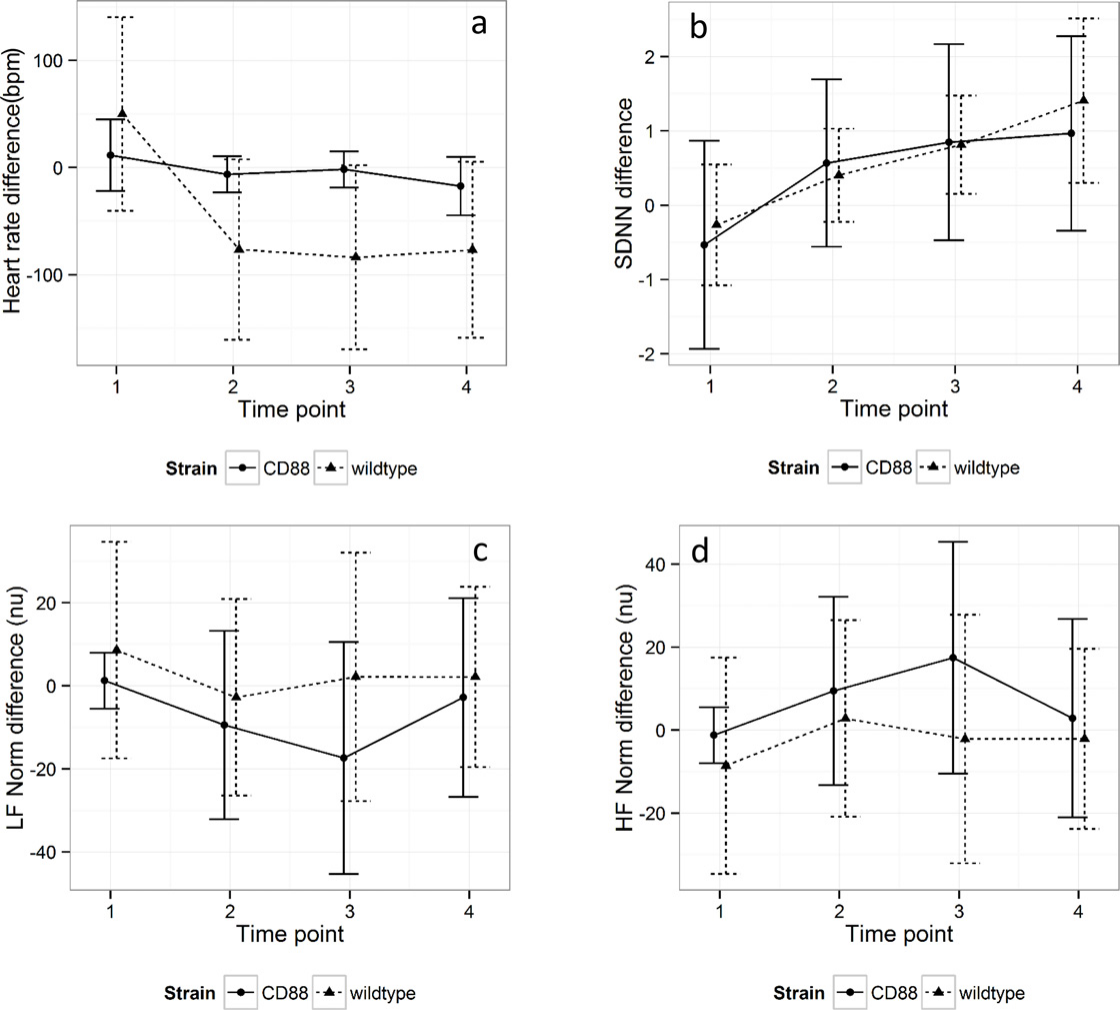
Difference in response to atenolol and placebo for (a) HR, (b) SDNN, (c) LF Norm and (d) HF Norm in WT and CD88-/- mice. Time point 1 represents the baseline prior to administration of drug (t = -30 min). Time points 2–4 represent experimental periods 30, 60 and 90 min, respectively. Error bars represent standard deviation. HR and LF Norm were constitutively lower and SDNN and HF Norm constitutively higher in the CD88-/- compared with WT mice (*P*<0.001 for all outcomes).

There was no difference in baseline HR, SDNN, LF Norm, HF Norm or LF/HF ratio for either the WT or the CD88-/- mice at the beginning of either the placebo or atenolol trial period, meaning that there was no effect of treatment order or time on the respective baseline parameters for the two groups of mice. However, for each model the baseline outcome measure was included to improve precision. There was no interaction between strain and time. Heart rate fell more in response to atenolol in the WT mice than in the CD88-/- mice, with a mean difference of 78.6 ± 23.6 bpm, *P* = 0.001. SDNN rose in the CD88-/- mice compared with the WT mice, with a mean difference of 63.8 ± 25.1, *P* = 0.01. There was no difference in response to atenolol between WT and CD88-/- mice for LF Norm, HF Norm or LF/HF ratio over time (Fig 4A–4D).

**Fig 5:**
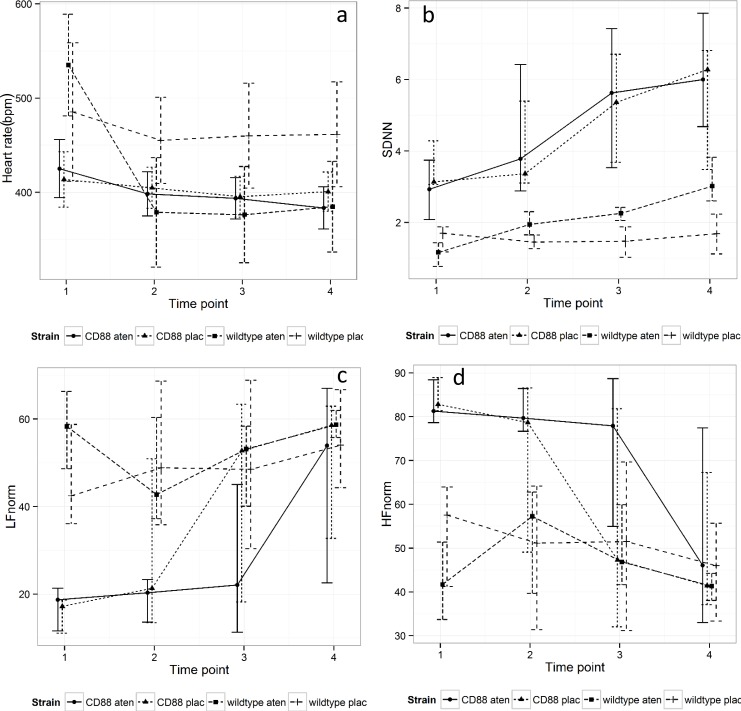
Changes in (a) HR, (b) SDNN, (c) LF Norm and (d) HF Norm for wild type (wt) and CD88-/- (cd) mice treated with placebo (plac) or atenolol (aten) over the time course of the experiment. Time point 1 represents the baseline prior to administration of drug (t = -30 min). Time points 2–4 represent experimental periods 30, 60 and 90 min, respectively. Error bars represent standard deviations.

HR and LF Norm were constitutively lower and SDNN and HF Norm constitutively higher in the CD88-/- compared with WT mice (*P*< 0.001 for all outcomes). Fig 5A–5D depicts the course of HR, SDNN, LF Norm and HF Norm over the time course of the experiment.

## Discussion

A major outcome from Study 1 was that administration of a C5aR1 antagonist drug (PMX53) attenuated the increased HR following reperfusion. Importantly, all the mice pre-treated with PMX53 survived the surgical intervention. The HR of the non-treated GutIR operated wild type mice began to decline towards the end of a 2.5 hr observation period and 5/9 mice were euthanased before the 5 hr mark due to marked clinical deterioration, meaning that further statistical comparisons between HR were meaningless. Ischemia, and subsequent reperfusion injury, is commonly associated with an inflammatory response and tissue damage [23,24,30] and may contribute to ongoing cardiac disease and, in this study, a marked reduction in survival rate was shown. Interestingly, there is some evidence that adrenergic receptors may be associated with inflammatory pathways, particularly the complement 5a (C5a) receptor [15] which, in itself, has been linked to cardiac disease [31] and, specifically, myocardial ischemia-reperfusion injury [22].

The link between inflammatory and adrenergic receptors was strengthened by the finding that absence of a C5aR1 had a major impact on the HR profiles, with wild type (normal) mice having a higher HR.The ANS, especially the sympathetic nervous system, plays a major role in regulating cardiovascular homeostasis, including during alterations in physiological status, such as during anesthesia, which is reflected as a variation in HRV [32]. There were no differences in the frequency-domain measures between the two strains of mice, yet anesthesia increased the sympathetic tone only in the knock-out mice, despite overall HR being lower than wild type mice. It would therefore appear possible that the C5a receptor may be important in the response to sympathetic activity during anesthesia and absence of this receptor in this type of knockout mouse disturbs the regulation of the cardiovascular sympathovagal tone or, alternatively, possibly disrupts the parasympathetic responses.

Analysis of HRV is typically performed using ECG and/or 24-hr Holter monitoring, which permit short-term (30–60 min) or long-term (up to 24 hr) recording respectively [11,33]. Implantable radiotelemetry [34,35] permits the collection of ECG data in unrestrained conscious mice to assess physiological [21,36], toxicological [37] or pharmacological interventions [27,38] on the autonomic control of the heart, although these methods have not been standardized [26]. In this present study, the published techniques to collect and analyze HR and HRV in conscious mice [19,20,26,39] were adapted for use in anesthetized mice. The advantage of the current model was that movement artifacts were reduced, permitting the detection of small but important changes in ECG that may otherwise be obscured [40].

A feature of the current study was the application of HRV analysis, which considers both time- and frequency-domains, as a non-invasive electrocardiographic marker of autonomic modulation of the HR, including the relative predominance of sympathetic or parasympathetic systems [11]. HRV reflects the dynamic interplay between the multiple physiologic mechanisms which regulate the instantaneous HR and R-R interval (intervals between QRS complexes of normal sinus depolarization) [11,12]. The electrical activity and contractile force of the myocardium is largely modulated by the ANS. In the normal physiological state, there is interplay between the sympathetic and parasympathetic outflows [12], while dysregulation of the ANS control of the heart may induce malignant dysrhythmias [10]. A decrease in HRV has been shown to reflect disturbances of the ANS that are related to increased cardiovascular dysfunction, including sudden cardiac death [41].

Atenolol will increase HRV and reduce the LF Norm component leading to reduced sympathetic activity in the hearts of healthy rodents [20,38], which is a useful outcome to control sympathetic nervous system hyperactivity in disease states [14]. In the current study, atenolol effectively attenuated HR over time, compared to baseline, which was consistent with earlier reports [33,42]. However, the reduction in HR was significantly greater in the WT mice, which could suggest that the C5aR1 may also be necessary for maximal response of the β_1_-AR to an agonist. This may also explain a smaller bradycardic effect of atenolol on the CD88-/- mice, even allowing for the lower initial baseline HR.

Furthermore, atenolol effectively increased HRV over time, in both wild type mice and CD88-/- mice. Again, maximal efficacy of the β_1_-AR antagonist appeared to also require the presence of the C5aR1, although HRV gradually increased over time, which suggested that the prolonged stressors (the anesthesia) attenuated the efficacy of β_1_-AR antagonist. These findings suggested that the effects of atenolol may diminish by 90 min, which is about the half-life of atenolol in the rat [43], and we assumed that it may be similar or shorter in mice, although we could not find any publications describing the pharmacokinetics of atenolol in mice.

Alternatively, the route of administration may affect atenolol activity in mice. Most values of the frequency domain measured in the wild type mice were not significantly different to baseline. However, graphically, the β_1_-AR antagonist (atenolol) post-intraperitoneal (fast-acting route) administration were observed to have reduced LF Norm, increased HF Norm and thus induced a lower LF/HF ratio at all time-points, compared to the placebo treatment group, suggesting predominant parasympathetic tone of the heart. Only at one time point (60 min post-atenolol) shown by the CD88-/- mice revealed that the LF/HF ratio was significantly reduced compared to the placebo treatment group. The non-significant observations were consistent with a study where healthy volunteers administered intravenous (acute β-blockade) did not modify the normalized component significantly, while oral administration (chronic β-blockade) significantly reduced the LF/HF ratio [42]. It would appear that any effects of atenolol over time should consider the pharmacokinetic profile in the target species or the β_1_-AR density. Of greater significance for the current study was that absence of the C5aR1 in mice increased HRV and reduced HR, which was similar to the effect of antagonizing β_1_-AR in the wild type mice (Study 2). Furthermore, the normal response to increase circulating plasma catecholamine activity in response to stress (Study 1), namely increased HR, is attenuated with absence of the C5aR1. It is uncertain if there is a physiological relationship between β_1_-AR and C5aR1, although it has been previously reported that C5aR interacts with the α-AR in the hypothalamus of rats [15]. However, we successfully demonstrated a possible association between β_1_-AR and CD88, but it is unclear if the association is direct or casual. The role of the complement system, particularly the biological function of C5a and its receptors (CD88 and C5L2) has been well characterized [44], but the role of the complement system in general, and C5a in particular in normal and diseased cardiovascular systems is not well understood and debate remains as to whether it plays a protective or deleterious role in specific disease states [45]. Decreased HRV observed in mice with over-expression of atrial β_1_-AR [46] would probably benefit from either β_1_-AR antagonist or a possible new therapeutic regime where antagonizing the C5aR, especially CD88, is an attractive strategy to treat and prevent a number of clinical conditions caused by excessive complement activation, especially in the heart.

### Limitations of the Study

Anesthesia was essential for this study, both to be able to undertake the surgical interventions and to reduce movement artefact during HRV assessment, but is also known to profoundly alter autonomic regulation. However, lower concentrations of isoflurane, such as in the current study, have been reported to induce a reflex increase in sympathetic tone [47], which may be considered further support to the findings in the current study that the C5aR is required for maximal responsiveness in the β1-AR. Furthermore, it should be recognized that HRV only provides a very indirect marker of autonomic regulation. Frequency domain analysis does not provide a quantitative assessment of sympathetic and parasympathetic balance [48], but was used in the current study to provide some index of autonomic activity over time. We also only had a limited availability of the CD88 knock-out mice, which were donated by a collaborator, whereas greater numbers in the groups may have increased statistical significance. Due to the limited number of the knock-out mice, we were not able to proceed with a GutIR operated CD88-/- mice for further observation of HR and HRV analysis which would be deemed useful to further support the evidence of association/interaction of β_1_-AR and C5aR1.

### Conclusion

We demonstrated the feasibility of using a quantitative analysis of beat-to-beat fluctuation in the healthy anesthetized mouse to assess the sympathetic and parasympathetic influences in HR modulation. Thus, application of a standardized HRV technique and study protocol of anesthetized mice should allow appropriate comparison among future studies typically in these two strains of mice. The methods would be useful to investigate the ANS system modulation in abnormal hearts in mouse models of disease such as myocarditis, ischemia and hypertrophy which has been associated as a potential underlying arrhythmogenic substrate. Here we demonstrated that both the β_1_-AR and C5aR1, specifically CD88-/-, indicated a possible association/interaction in the mice myocardium. This study also suggests that looking at the receptors in-vivo studies is crucial. Expression of the receptors in the healthy mice versus mice subjected to reperfusion injury upon pre- and post- treatment using atenolol and PMX53 is crucial as a further study.
